# Brain Age Prediction of Children Using Routine Brain MR Images via Deep Learning

**DOI:** 10.3389/fneur.2020.584682

**Published:** 2020-10-19

**Authors:** Jin Hong, Zhangzhi Feng, Shui-Hua Wang, Andrew Peet, Yu-Dong Zhang, Yu Sun, Ming Yang

**Affiliations:** ^1^School of Informatics, University of Leicester, Leicester, United Kingdom; ^2^Department of Radiology, Children's Hospital of Nanjing Medical University, Nanjing, China; ^3^School of Architecture Building and Civil Engineering, Loughborough University, Loughborough, United Kingdom; ^4^School of Mathematics and Actuarial Science, University of Leicester, Leicester, United Kingdom; ^5^Institute of Cancer & Genomic Science, University of Birmingham, Birmingham, United Kingdom; ^6^Department of Information Systems, Faculty of Computing and Information Technology, King Abdulaziz University, Jeddah, Saudi Arabia; ^7^International Laboratory for Children's Medical Imaging Research, School of Biology Science and Medical Engineering, Southeast University, Nanjing, China

**Keywords:** magnetic resonance imaging, deep learning, brain age, convolutional neural network, artificial intelligence

## Abstract

Predicting brain age of children accurately and quantitatively can give help in brain development analysis and brain disease diagnosis. Traditional methods to estimate brain age based on 3D magnetic resonance (MR), T1 weighted imaging (T1WI), and diffusion tensor imaging (DTI) need complex preprocessing and extra scanning time, decreasing clinical practice, especially in children. This research aims at proposing an end-to-end AI system based on deep learning to predict the brain age based on routine brain MR imaging. We spent over 5 years enrolling 220 stacked 2D routine clinical brain MR T1-weighted images of healthy children aged 0 to 5 years old and randomly divided those images into training data including 176 subjects and test data including 44 subjects. Data augmentation technology, which includes scaling, image rotation, translation, and gamma correction, was employed to extend the training data. A 10-layer 3D convolutional neural network (CNN) was designed for predicting the brain age of children and it achieved reliable and accurate results on test data with a mean absolute deviation (MAE) of 67.6 days, a root mean squared error (RMSE) of 96.1 days, a mean relative error (MRE) of 8.2%, a correlation coefficient (*R*) of 0.985, and a coefficient of determination (*R*^2^) of 0.971. Specially, the performance on predicting the age of children under 2 years old with a MAE of 28.9 days, a RMSE of 37.0 days, a MRE of 7.8%, a *R* of 0.983, and a *R*^2^ of 0.967 is much better than that over 2 with a MAE of 110.0 days, a RMSE of 133.5 days, a MRE of 8.2%, a *R* of 0.883, and a *R*^2^ of 0.780.

## Introduction

The brain development of children undergoes a rapid and complex process, especially in the first 2 years after birth (1, 2). The early brain development follows the law of myelination from caudal to rostral, posterior to anterior regions, central to peripheral locations, which is closely related to the development of sensory, motor, and cognitive ability (3). Delayed brain development can lead to intellectual disability, language disorder, activity limitation, and other manifestations in children, which seriously affect their quality of life. Therefore, accurate and quantitative evaluation of brain development, early identification, and intervention treatment is particularly important for children with brain development analysis and brain disease diagnosis.

At present, brain magnetic resonance (MR) imaging is a reliable method to evaluate brain development (brain age) due to its non-invasive, high soft tissue resolution and multi-parameter imaging advantages. Recently, the main ways of MR image to evaluate brain development are as follows: morphometry [including measurement of brain volume (4–6), cortical thickness (7), surface area (7, 8), etc.], white matter diffusion (9, 10), functional connectivity (11–14). However, there are some drawbacks within these studies: the need of some special sequences with long scanning time, complex data post-processing, and group-level comparison results without quantitative analysis to individuals, which limit their wide use in clinical situations.

With the development of deep learning, more and more sophisticated deep neural networks have been proposed to analysis massive image, voice, or video data. Of these, convolutional neural network (CNN) of deep learning has achieved great success with superior performance beyond human experts in many computer vision and speech recognition tasks since it was put forward (15–20). In the field of medical image analysis, CNN-based method has been also proposed for disease diagnosis and lesion detection with high performance in accuracy, such as the classification and detection of lung nodules (21, 22), the recognition of melanoma (23), the detection of cerebral microbleeds (24–26), as well as the classification of Alzheimer's disease (27, 28). In addition, brain age prediction based CNN model has been proved to be a reliable and heritale biomarker of brain aging and can be used to indicate the risk of brain degenerative diseases (29, 30), whereas it has not been reported in young children up to now. Furthermore, unlike traditional machine learning approaches that implement feature extraction, feature reduction, and classification separately, CNN combines them as an end-to-end system, from raw images to the corresponding target values, avoiding complicated image preprocessing and manual design of appropriate features. The excellent performance and transferability lead us to believe that CNN-based method should be the most promising resolution for most clinical applications, including brain age prediction of children.

In this paper, we collected 220 routine brain MR images of healthy children for investigating the brain age of children based on deep learning. Data augmentation was utilized to extend the training data for avoiding the potential over-fitting and enhancing generalizability of the model. With delicate design of structure and careful setting of hyper parameters, we proposed a 3D deep neural network and achieved high performance. We analyzed the prediction results of different age groups in detail and compared them with those of other two state-of-the-art methods. The factors in the proposed model that may affect the prediction results were investigated comprehensively. Furthermore, we compared the proposed 3D CNN with the corresponding 2D CNN that has a similar structure in predicting brain age of children with 3D MR image data.

## Methods

### Dataset Acquisition

Ethical approval for the research was obtained from the ethics committee of Children's Hospital of Nanjing Medical University. This is a retrospective study, and informed written consent was thus waived. The dataset consists of T1-weighted images of 220 healthy children aged 0 to 5 years old. The data were all acquired using a 1.5T Siemens Avanto Scanner, but scanning parameters of newborns (≤ 1 month) are different from older children due to variation in water content of brain tissue. Scans of newborns were imaged using a T1-weighted spin-echo sequence (repetition time [TR] = 4,490 ms, echo time [TE] = 7.5 ms, flip angle [FA] = 150°, 18 slices, slice thickness = 4.5 mm, FOV = 180 × 180 mm, voxel dimensions = 1.0 × 0.7 × 4.5 mm). Scans of older children (>1 month) were also imaged using the T1-weighted spin-echo sequence (TR = 3,850 ms, TE = 7.3 ms, FA = 150°, 22 slices, slice thickness = 5.0 mm, FOV = 220 × 220 mm, voxel dimensions = 1.4 × 1.0 × 5.0 mm). Those whose brain MR image quality was good enough to diagnose and reports were diagnosed as normal by two experienced radiologists, and whose history, clinical data, and phone call following-up can't show the existence of neurological disease were enrolled into our dataset. Premature infants, subjects who were diagnosed with congenital diseases (congenital heart diseases, Down's syndrome, etc.), neurodevelopmental or mental disorders (neurodevelopmental delay, autism, etc.), and other serious illnesses (hypoxic-ischemic encephalopathy, cerebral hemorrhage, septicopyemia, etc.) affecting brains were excluded from our dataset. Furthermore, we used downsampling method to convert the stacked 2D brain MR images of newborns and older children to the same size of 128 × 116 × 12.

### Data Augmentation Technology

Basically, data augmentation methods are extensively used to train a deep neural network having huge parameters for improving prediction accuracy that had been validated in the “Results” section. In our experiments, four commonly used methods of data augmentation were employed to enhance the training dataset. They are listed as: (a) scaling, (b) image rotation, (c) translation, and (d) gamma correction. We scaled images with scaling factor of 0.85 to 1.15 with step of 0.03 for generating 10 new images. Image rotation was used to generate 10 new images with rotation angle of −15 to 15 degrees increased by 3 degrees. We translated images with factor of −0.1 to 0.1 with step of 0.02 diagonally for generating 10 new images. Gamma correction with gamma value of 0.85 to 1.15 increased by 0.03 was employed to generate 10 new images. At last, we augmented the training dataset by 41 times using data augmentation methods.

**Figure 1:**
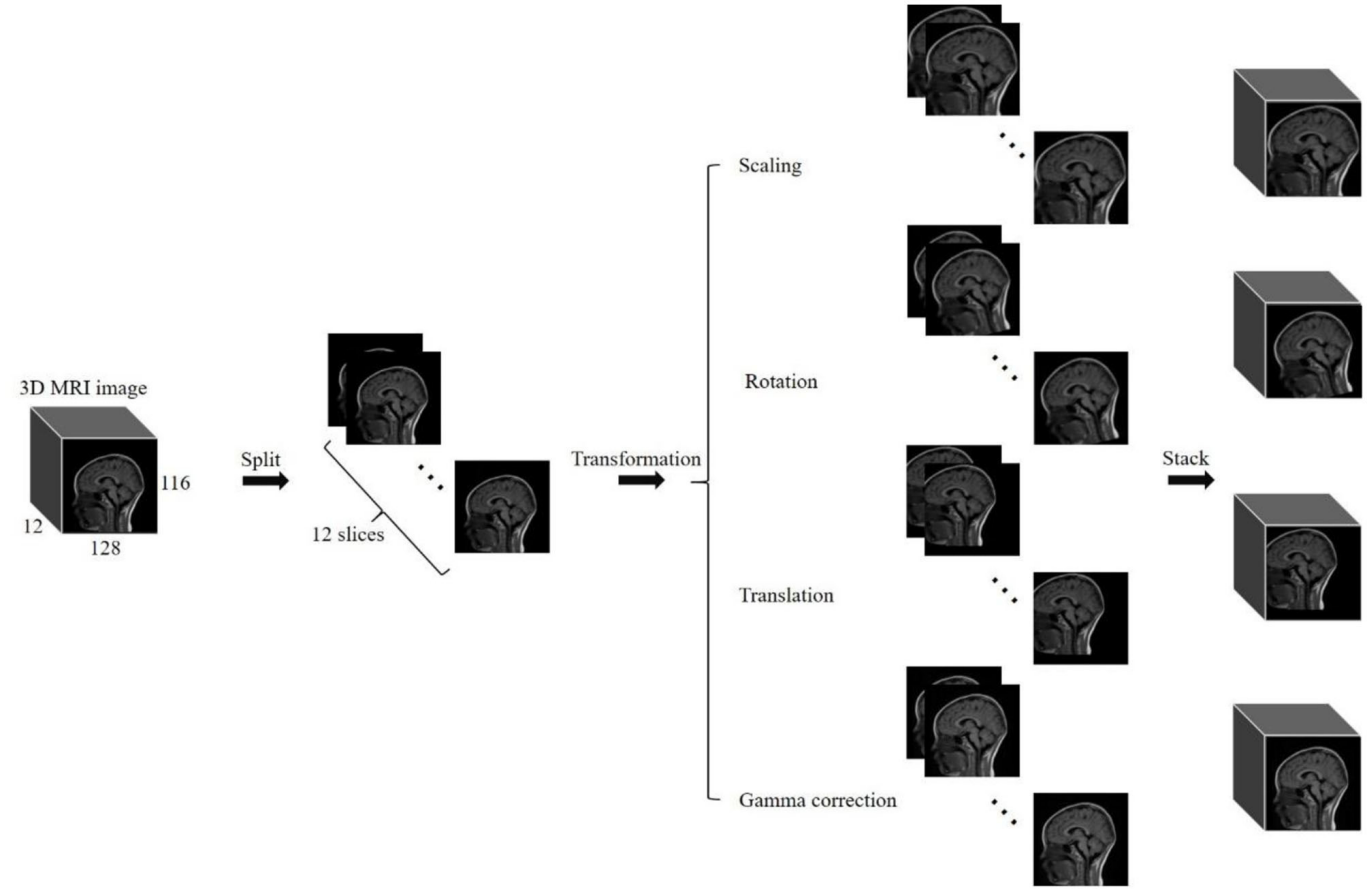
Illustration of data augmentation to a 3D image.

Noted: For one routine brain MR image with size of 128 × 116 × 12 in this paper, we split the volume into 12 slices, then used the same transformation method to process every slice, and finally stacked those slices into a 3D image (see Figure 1). We achieved the transformation of the whole 3D image by this way.

### Proposed 3D CNN Architecture

**Figure 2:**
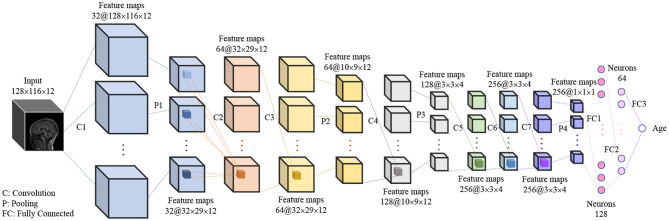
The hierarchical architecture of proposed 3D CNN. The “32” and “128 × 116 × 12” in “32@128 × 116 × 12” denote the number and size of feature maps.

A 3D CNN was proposed to predict the brain age of children using brain MR images with size of 128 × 116 × 12. The 3D image was input to the model and then a single scalar denoting the brain age was output. The proposed 3D CNN model, shown in Figure 2, contains 7 3D convolution layers, 4 3D max pooling layers, and 3 fully connected layers. All convolution layers are followed by 3D batch-normalization (31) and ReLU activation function (32), while the first two fully connected layers are followed by ReLU activation function. All convolution layers have the same kernel size of 3 × 3 × 3, stride size of 1 × 1 × 1, and padding size of 1 × 1 × 1, which means the feature map size is the same as that of the input. The kernel size in the first, second, third, and fourth max pooling layer are 4 × 4 × 1, 3 × 3 × 1, 3 × 3 × 3, and 3 × 3 × 3, respectively, and the stride size is equal to the kernel size in all max pooling layers.

The mean absolute error (MSE) was used as the loss function. The reliable and commonly used stochastic gradient descent with momentum of 0.9 (SGDM) was employed as the optimization method. The mini-batch and epoch were set to 64 and 40, respectively. The initial learning rate was set to 0.0000008 and decreased by 10% every epoch. The weights were initialized randomly.

Note: 10 runs were implemented for accounting for the stochastic properties of CNN, and the average value of the 10 runs is regarded as the final result.

### 2D CNN Architecture

**Figure 3:**
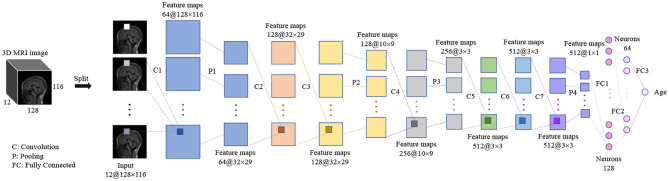
The hierarchical architecture of the 2D CNN.

We designed a 2D CNN model, shown in Figure 3, according to the structure of the proposed 3D CNN, so both have similar hierarchical structures. The 3D image with size of 128 × 116 × 12 was split into 12 slices, and those slices were then input to the 2D CNN model, and finally the age was given. Same as the proposed 3D CNN, all convolution layers in 2D CNN are followed by batch normalization and ReLU, and the first two fully connected layers are followed by ReLU. The kernel size, stride size, and padding size in convolution layers are 3 × 3, 1 × 1, and 1 × 1, respectively. The kernel size is equal to stride size in all max pooling layers, and they are 4 × 4, 3 × 3, 3 × 3, and 3 × 3, respectively, from the first to the last max pooling layer. In terms of hyperparameters, the loss function, optimizer, mini-batch, epoch, learning rate, and weights initialization were set the same as the proposed 3D CNN.

### Software Availability and PC Configuration

All data augmentation methods were implemented using imgaug (https://github.com/aleju/imgaug). All experiments of deep learning were carried out on PyTorch (https://pytorch.org/). The running environment of the programs: i9-9900k CPU, NVIDIA GeForce RTX 2080 Ti GPU, and 16.0 GB RAM.

## Results

### Dataset Characteristics

**Figure 4:**
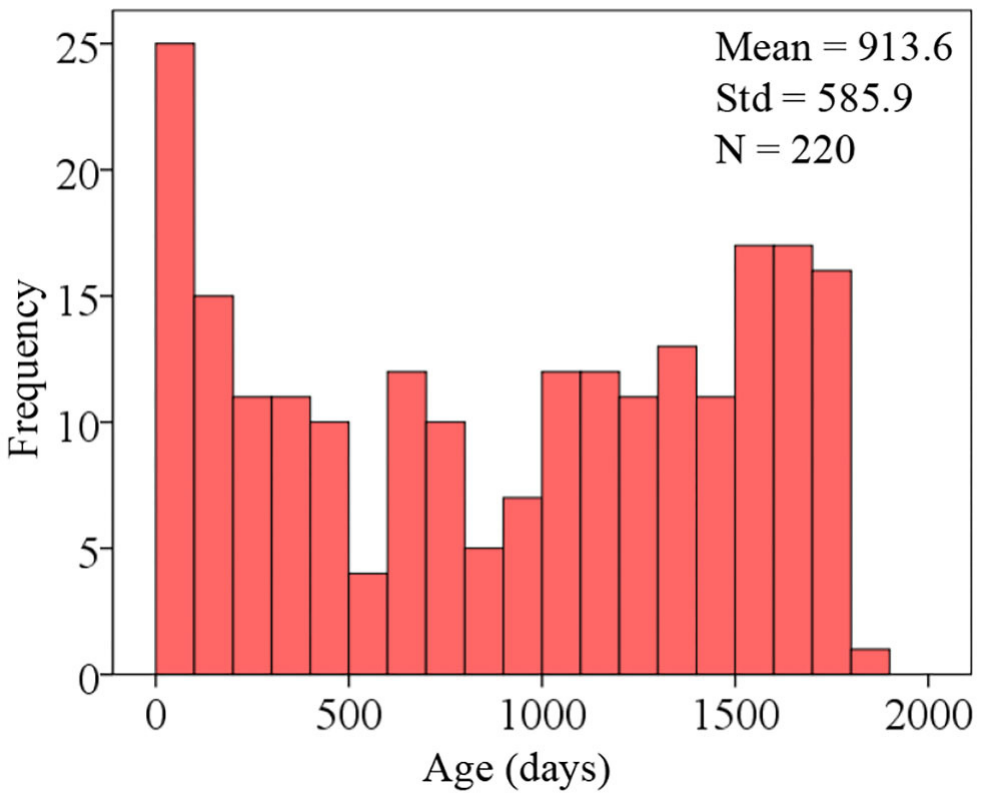
Distribution of participant ages.

To develop an AI system for predicting the brain age of children using routine clinical brain MR images, we enrolled 220 subjects aged 0 to 5 years old (Figure 4 shows the distribution of participant ages with 100-day intervals) and scanned them to achieve the brain MR images. The hold-out method was employed to divide the 220-image dataset into two parts randomly, and one part containing 176 images (80%) was regarded as training dataset and the other part containing 44 images (20%) as test dataset. The reason for abandoning the validation dataset is that the whole dataset only contains 220 subjects. Table 1 gives the demographic information of the training and test datasets. Since the amount of the training dataset is slightly small to train a deep neural network, data augmentation was implemented for generating new “fake” images. At last, the training dataset containing 7,216 images and the test dataset containing 44 images were obtained.

**Table 1:** Subjects demographic (Std denotes standard deviation).

	**Total dataset**		**Test dataset**		**Training dataset**	
	**0–2 years old**	**2–5 years old**	**0–2 years old**	**2–5 years old**	**0–2 years old**	**2–5 years old**
Subjects	88	132	23	21	65	111
Age (days)	4–697	731–1820	36–680	749–1687	4–697	731–1820
Mean **±** Std	283.6 ± 215.7	1333.5 ± 314.2	244.2 ± 201.0	1267.0 ± 288.4	297.6 ± 220.5	1346.1 ± 318.4

### Performance of the Proposed Model

With grid search and trial-and-error methods, we optimized a 3D CNN model for predicting the children's age more accurately and reliablly. The detailed information of the proposed model can be found in Figure 2. The model was trained by the data-augmented dataset including 7,216 images and evaluated on the test dataset including 44 images. In our experiments, the learnable weights of model were initialized randomly, and the random seed was not fixed, causing the randomness of prediction results. Thus, we implemented 10 runs under the same settings of the model for ensuring the reliability of the results.

**Figure 5:**
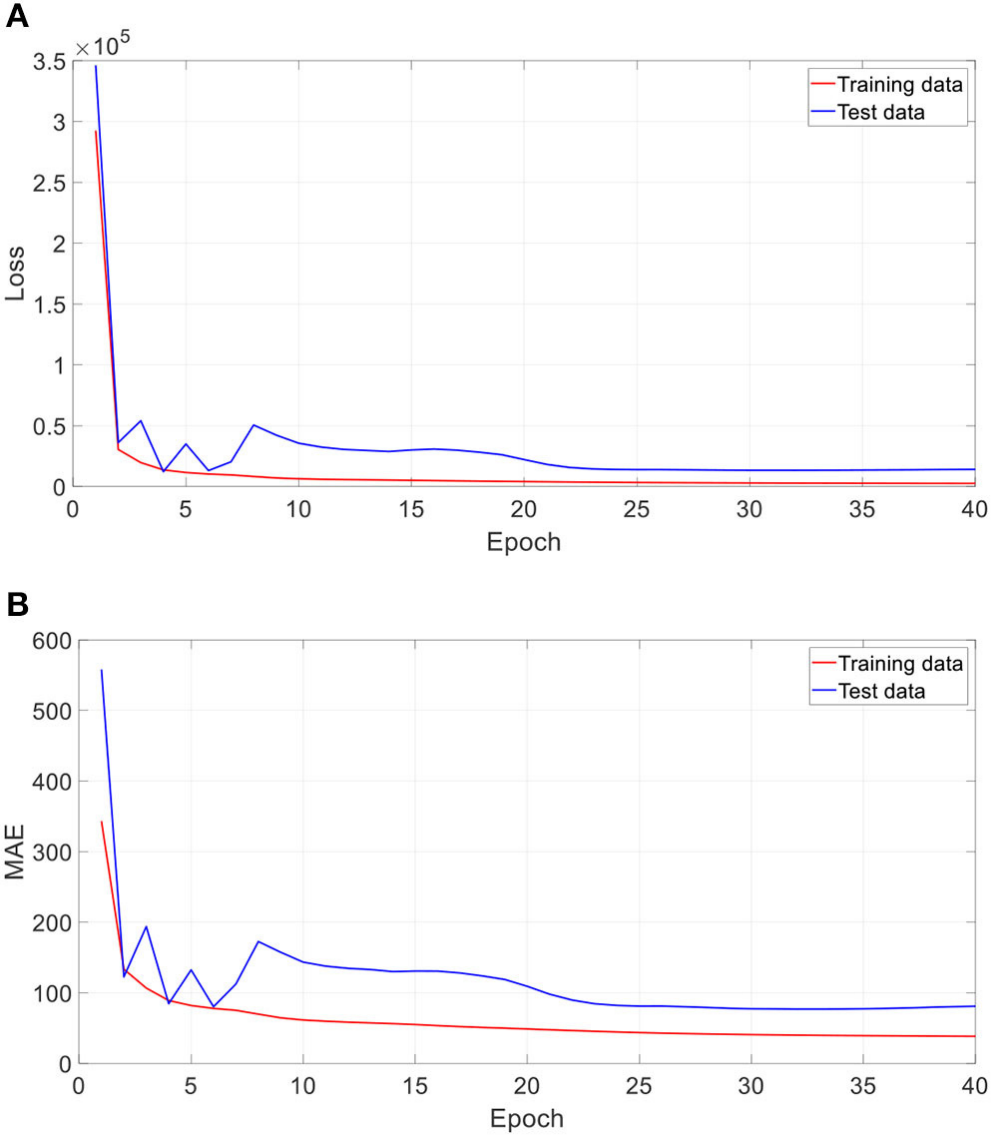
Training performance of one run. **(A)** Loss against training epoch, and **(B)** MAE against training epoch. The loss and MAE are the average of all iterations in one epoch.

Figure 5 shows the training performance of one typical run. As Figure 5 shows, after 40 epochs (iterations through the whole training dataset), both training dataset and test dataset in loss and MAE reached a plateau and were at a minimum, which means that the training process has converged.

**Figure 6:**
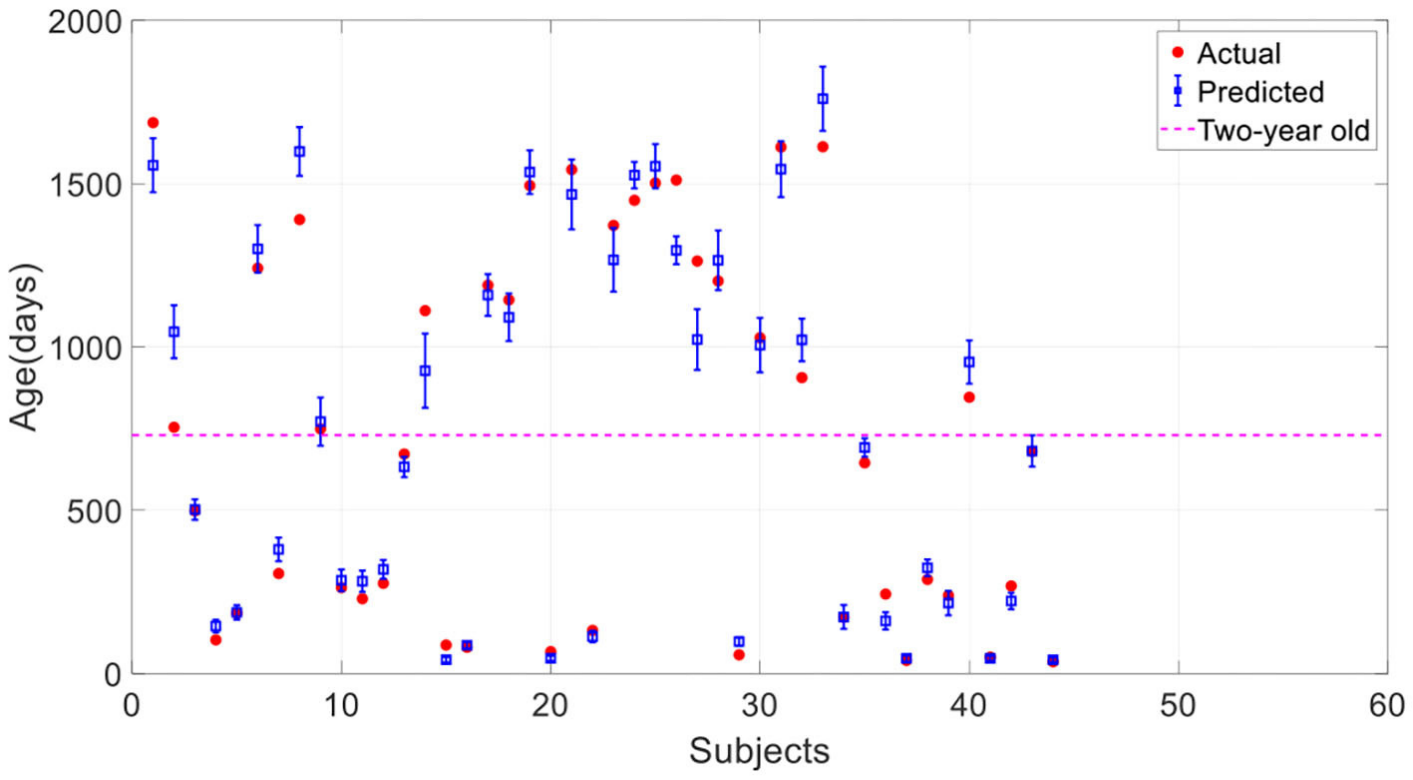
Prediction results of the proposed 3D CNN. The error bar represents the average and standard deviation of the prediction results over 10-run.

Figure 6 shows the average and standard deviation of prediction results of 10 runs under the same setting. It is found that most true data fall within the standard deviation of predicted data, which means that the predictions can fit the true data well. To further quantitatively evaluate the prediction accuracy of the model, MAE, RMSE, MRE, *R*, and *R*^2^ between the average values and the true values were employed (Table 2). With a MAE of 67.6 days, a RMSE of 96.1 days, a MRE of 8.2%, a *R* of 0.985, and a *R*^2^ of 0.971, the proposed model was considered to achieve quite high accuracy in predicting the brain age of children aged 0 to 5 years old.

**Table 2:** Performance of the proposed 3D CNN in predicting children aged.

**Age group (years old)**	**MAE (days)**	**RMSE (days)**	**MRE (%)**	***R***	***R*^**2**^**
0–2	28.9	37.0	7.8	0.983	0.967
2–5	110.0	133.5	8.2	0.883	0.780
0–5	67.6	96.1	8.2	0.985	0.971

Since the brain development of infants under 2 years old is heterogeneous and particularly rapid, it is necessary to divide the age into two age groups according to 2 years old and evaluate the prediction results of the two groups separately. Table 2 gives the assessment results. We found that age predictions for children under 2 years old are significantly better than those over two according to all evaluation indicators. We can also observe that most predictions under 2 years old are closer to the true values compared with those over 2 years old in Figure 6. Comparing to the 0–2 age group, there is a stronger correlation between predicted and true values in the age group from 0 to 5 years old according to *R*, but there is a bigger MAE. The reason is that the true values in 0–2 age group are smaller as a whole than that in 0–5.

**Figure 7:**
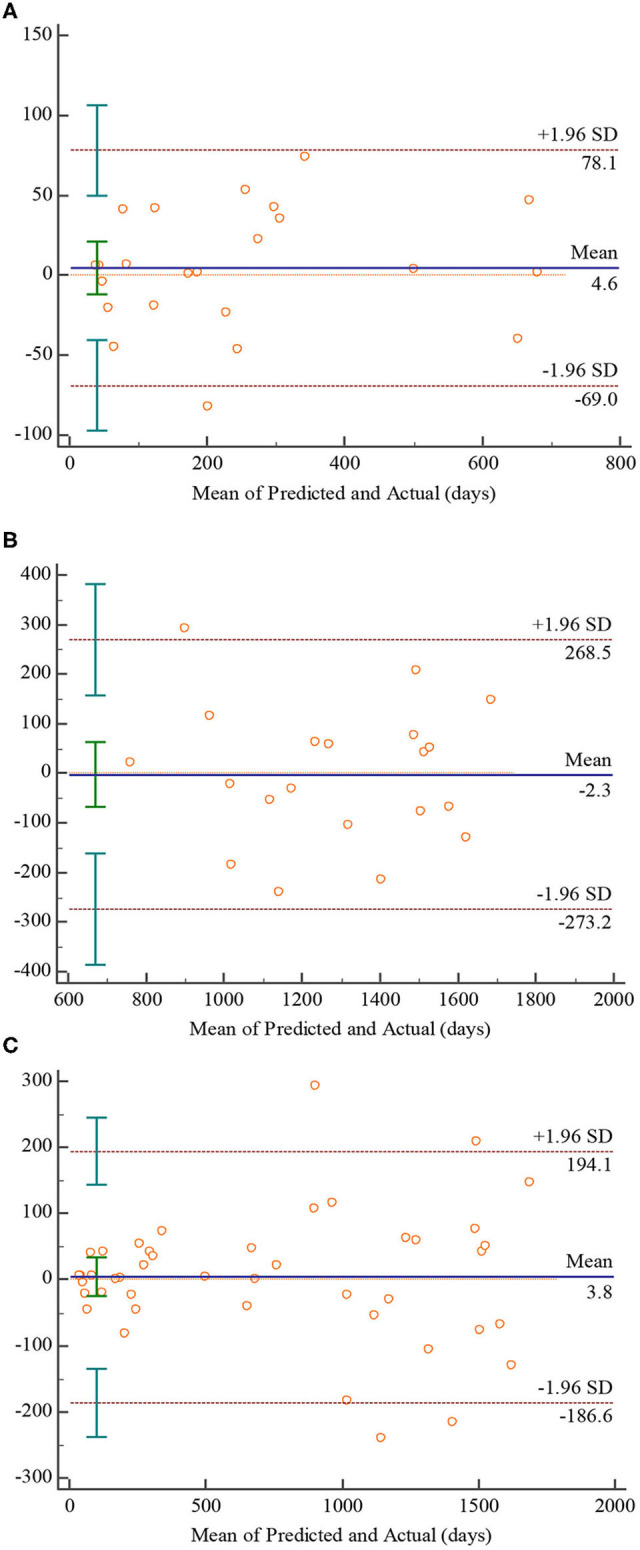
Bland-Altman plots for the proposed 3D CNN. Plot **(A–C)** denote 0–2, 2–5, 0–5 age groups, respectively.

To further assess the reliability of the predicted results, we gave the residual plot (Bland-Altman plot) and performed paired samples *T*-test. The residual plot was employed to show the relationship between mean and difference of the predicted and actual value, which is show in Figure 7. The *P*-values of the paired samples *T*-test were 0.5665, 0.9407, and 0.7979 in 0–2, 2–5, and 0–5 age groups, respectively, showing that there are no significant statistical differences between the predicted and the actual values of all age groups. As the Figure 7A indicating the 0–2 age group shows, 95.7% (22/23) of the points fall within the 95% limits of agreement, and the mean of difference is 4.6, which is close to 0. Similar to the 0–2 age group, 95.2% (20/21) of the points fall within the 95% limits of agreement, and the mean of difference is −2.3 according to Figure 7B. In terms of 0–5 age group, which is shown in Figure 7C, 90.9% (40/44) of the points fall within the 95% limits of agreement, and the mean of difference is 3.8 which is quite close to 0.

### Impact of Data Augmentation

In this research, the amount of the obtained 220-subject dataset is big enough to draw a conclusion of statistical analysis, but it is still insufficient for training a deep neural network with huge parameters. Many researches have reported that increasing the number of samples in training data can avoid over-fitting, enhance generalizability, and improve the performance on test set (33–37). Therefore, we performed data augmentation on a 176-subject training dataset and extended the dataset to 7,216 in our proposed method.

**Figure 8:**
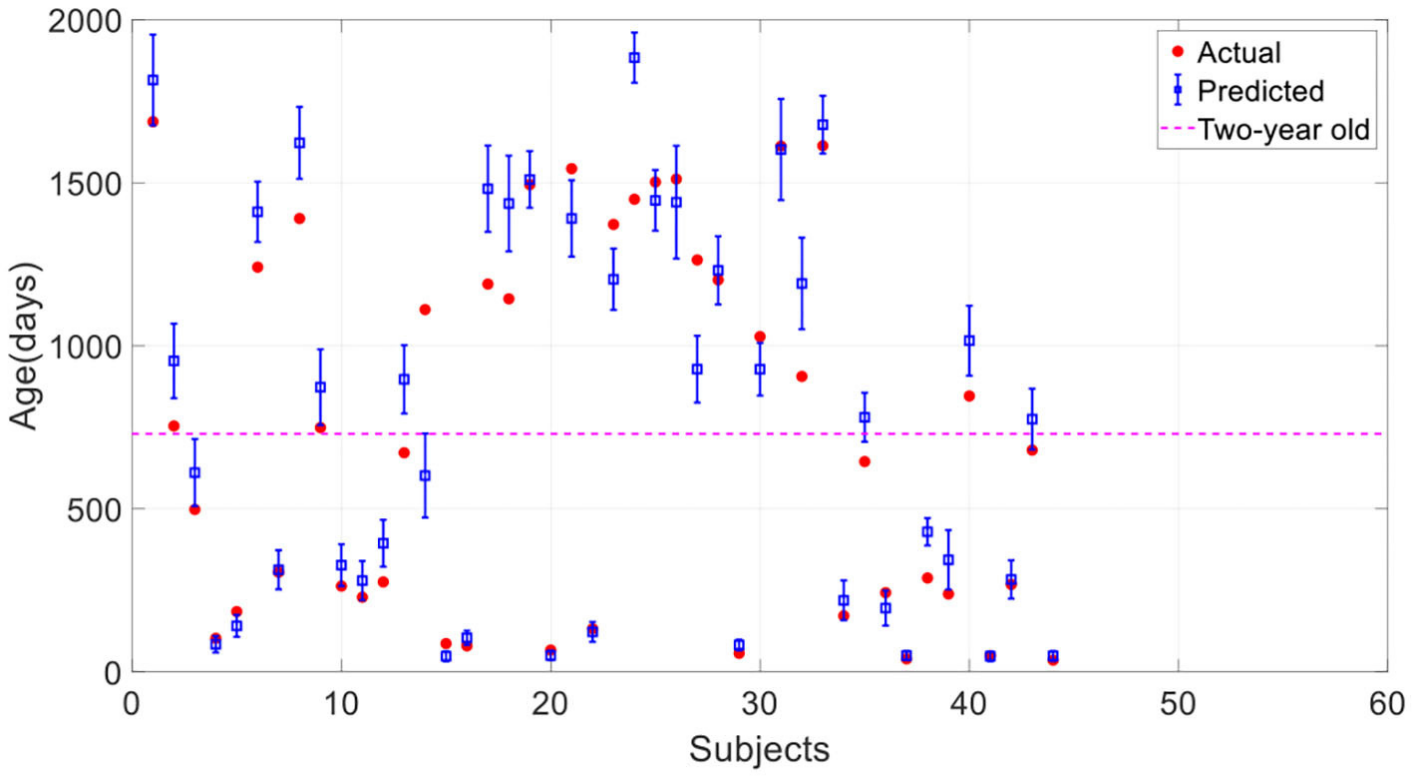
Prediction results of the proposed method without data augmentation. The error bar represents the average and standard deviation of the prediction results over 10-run.

Here we investigated the impact of data augmentation on predicting children's age using stacked 2D routine clinical brain MR images. Figure 8 offers the prediction results of our proposed method without data augmentation. All the results are average on 10 runs. Comparing with Figure 6, it is found that most of the predicted values deviate from the true values further, and the standard deviation of the predicted values is larger, showing the instability of the model. Table 3 gives a detailed comparison of our proposed method with and without data augmentation, which further confirms data augmentation can improve the prediction accuracy of the model.

**Table 3:** Comparison of the proposed method with and without data augmentation.

	**MAE (days)**	**RMSE (days)**	**MRE (%)**	***R***	***R*^**2**^**
Without data augmentation	118.3	166.7	13.7	0.963	0.926
With data augmentation	67.6	96.1	8.2	0.985	0.971

### Impact of Network Depth

**Figure 9:**
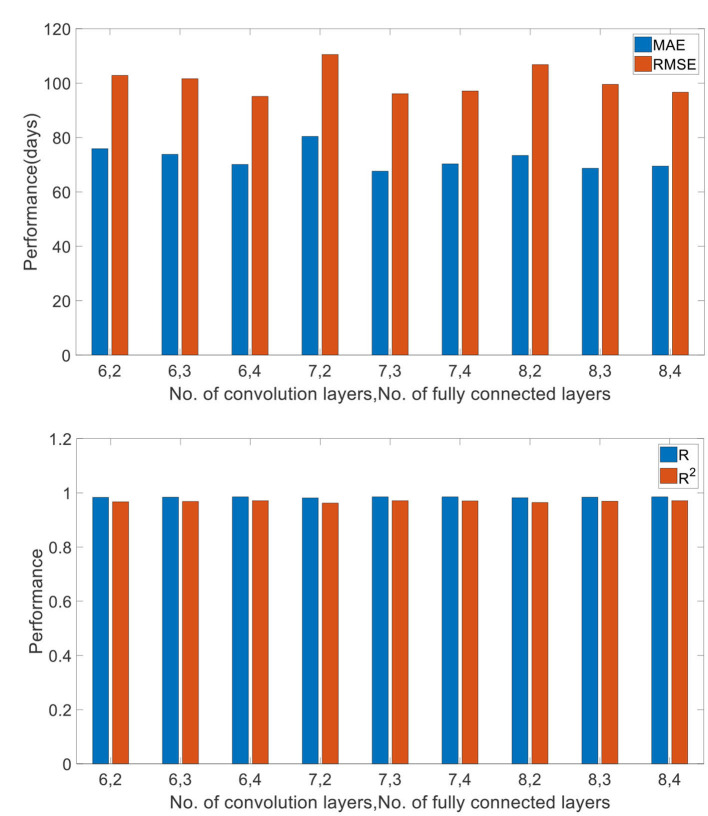
Comparison of different network depths. “6, 2” (“No. of convolution layers, No. of fully connected layers”) denotes 6 convolution layers and 2 fully connected layers.

To test the impact of network depth on performance of predicting children age, different 3D CNN including different convolution layers and fully connected layers were validated. The evaluation results are given in Table 4. Ten runs were implemented, and the average values were regarded as the final results. It is found that the proposed 3D CNN structure containing seven convolution layers and three fully connected layers achieved the best performance according to the comprehensive assessment of four indicators. Figure 9 is utilized to further visualize the performance differences between different 3D CNN.

**Table 4:** Performance of different network depths.

**No. of convolution layers**	**No. of fully connected layers**	**MAE (days)**	**RMSE (days)**	**MRE (%)**	***R***	***R*^**2**^**
6	2	75.9	102.8	9.2	0.983	0.967
6	3	73.8	101.6	9.0	0.984	0.968
6	4	70.1	95.1	8.4	0.985	0.971
7	2	80.4	110.5	10.0	0.981	0.962
7	3	67.6	96.1	8.2	0.985	0.971
7	4	70.3	97.1	8.6	0.985	0.970
8	2	73.4	106.8	9.2	0.982	0.964
8	3	68.7	99.5	8.2	0.984	0.969
8	4	69.5	96.6	8.3	0.985	0.971

### Impact of Batch Normalization

**Figure 10:**
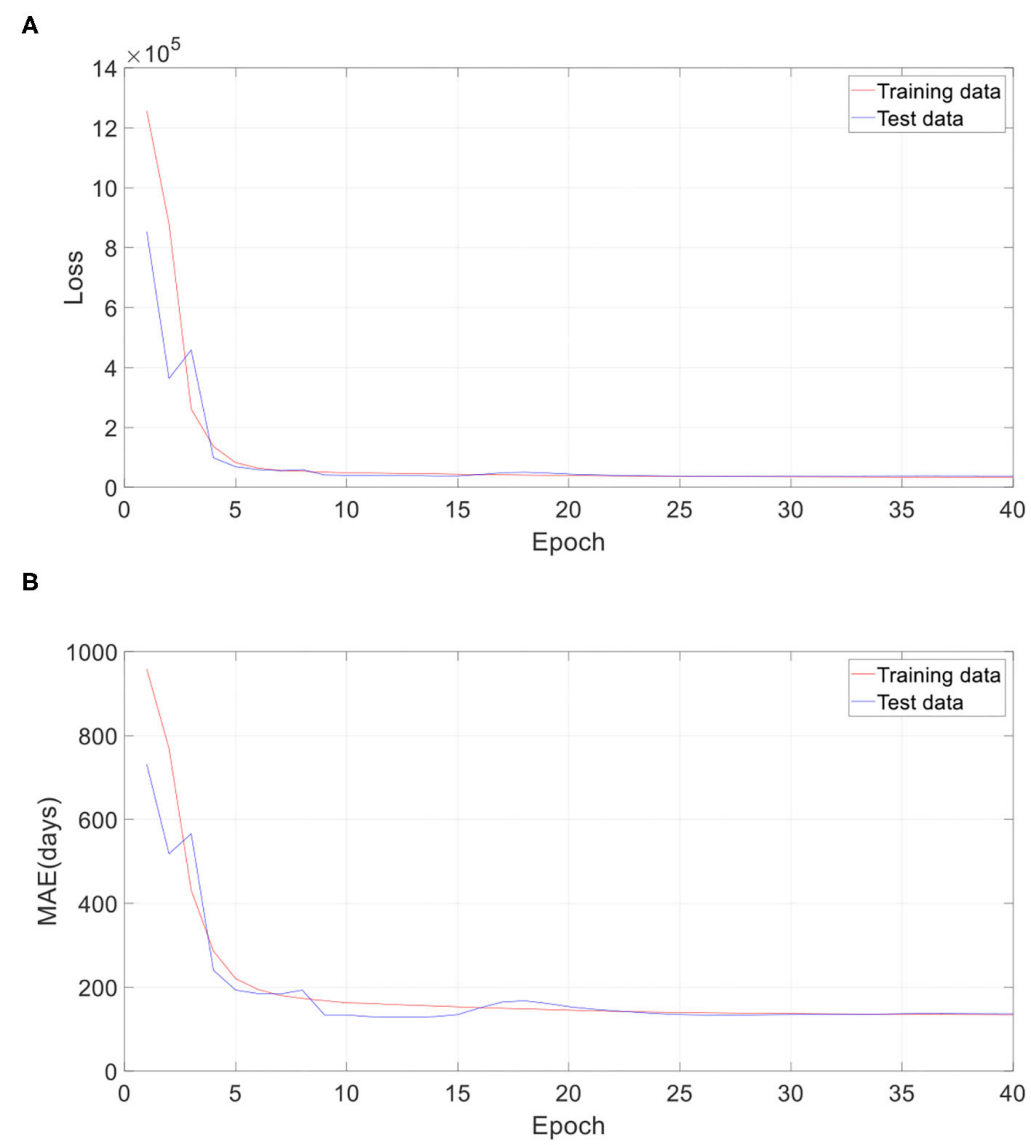
Training performance of one run without batch normalization. **(A)** Loss against training epoch, and **(B)** MAE against training epoch. The loss and MAE are the average of all iterations in one epoch.

In the proposed 3D CNN structure, every 3D convolution layer is followed by a 3D batch normalization. We investigated the impact of batch normalization on prediction accuracy in this section. All batch normalization layers were removed, and initial learning rate was set as 0.000000008. All other settings remain the same. Table 5 gives the comparison result of the proposed approach with and without batch normalization. All the results are averaged on 10 runs. As Table 5 shows, the 3D CNN without batch normalization achieved a MAE of 132.6 days, a RMSE of 189.9 days, a MRE of 15.0%, a *R* of 0.945, and a *R*^2^ of 0.893, which is obviously worse than the proposed 3D CNN with batch normalization. Figure 10 gives the training performance of the 3D CNN without batch normalization. As we can see, both training dataset and test dataset in loss and MAE reached the minimum plateau, indicating that the network is fully trained.

**Table 5:** Comparison of the proposed method with and without batch normalization.

	**MAE (days)**	**RMSE (days)**	**MRE (%)**	***R***	***R*^**2**^**
Without batch normalization	132.6	189.9	15.0	0.945	0.893
With batch normalization	67.6	96.1	8.2	0.985	0.971

### Impact of Batch Size and Learning Rate

Except for the structure, hyper parameters also can affect the 3D CNN performance. We compared different prediction results of the proposed 3D CNN trained by different batch size and initial learning rate for understanding the influence of them on the performance. Table 6 gives the survey results. All results are average on 10 runs. As the Table 6 shows, the 3D CNN with batch size of 64 and learning rate of 0.0000008 achieved the best prediction results according to all four evaluation indicators.

**Table 6:** Comparison of the proposed 3D CNN trained by different batch size and initial learning rate.

**Batch size**	**Learning rate**	**MAE (days)**	**RMSE (days)**	**MRE (%)**	***R***	***R*^**2**^**
16	0.0000008	72.3	96.2	8.3	0.985	0.971
32	0.0000008	74.1	97.4	9.2	0.985	0.971
64	0.0000008	67.6	96.1	8.2	0.985	0.971
64	0.0000012	69.9	97.1	8.6	0.985	0.971
64	0.0000004	75.0	104.8	9.3	0.983	0.966

### Comparing With 2D CNN

The input of 2D CNN is a 2D image with three color channels (i.e., RGB) in most natural scenes. With this regard, the simplest way for 2D CNN to deal with 3D input is to replace the color channels with the slices of the volumetric image. We designed a 2D CNN model, shown in Figure 3, according to the architecture of the proposed 3D CNN model for predicting the brain age of children using stacked 2D routine clinical brain MR image (gray-level) and investigated the performance differences between the two models. The comparison results are given in Table 7. All the results are average on 10 runs. We observed that the proposed 3D CNN achieved better performance in terms of all the evaluation indicators.

**Table 7:** Comparison of 2D CNN and our proposed 3D CNN.

	**MAE (days)**	**RMSE (days)**	**MRE (%)**	***R***	***R*^**2**^**
2D CNN	75.1	104.6	9.2	0.982	0.965
3D CNN	67.6	96.1	8.2	0.985	0.971

## Discussion

### High Reliability and Accuracy of 3D CNN for Brain Age Prediction

It is important to predict the brain age reliably and accurately for brain development analysis and brain disease diagnosis in pediatric patients. Basically, methods for predicting brain age can be divided into two categories: shallow learning algorithms and deep learning algorithms (38). So far, numerous shallow learning algorithms have been developed, such as gaussian processes regression (GPR) (29, 39, 40), support vector regression (SVR) (41, 42), partial least squares (PLS) regression (43), relevance vector regression (RVR) (44), hidden Markov model (HMM) (45), and Bayesian linear discriminant analysis (46). In terms of deep learning algorithms, CNN (29, 47) and back propagation neural network (BPNN) (48) were proposed to predict the brain age with brain MR images.

As the above references report, for achieving fairly good prediction result, all methods except CNN need to accomplish the complicated preprocessing task well including feature selection, dimension reduction, and segmentation of brain MR image into gray matter (GM), white matter (WM), and cerebrospinal fluid (CSF) tissues. The manual interventions in preprocessing lead to high intra-observer and inter-observer variability, which easily biased the final interpretation. Comparing to the traditional machine learning methods, CNN-based methods are an end-to-end system that uses the raw MR image data as the input and output the age value without manual interventions, showing higher reliability and improving clinical practice (38).

Although there is no error caused by manual intervention in predicting brain age using CNN-based model, there may be systematic bias (49, 50). As reported in (49), CNN-based model will overestimate the younger and underestimate the older, decreasing the reliability of prediction results. To evaluate the reliability of the predicted results in this paper, the Bland-Altman plots characterizing the relationships between the mean and the difference of the predicted and actual value were given, showing in Figure 7. According to Figures 7A,B, the mean of difference in the 0–2 age group is slightly higher than 0, while that in the 2–5 age group is slightly lower than 0. This observation seems to indicate that the prediction results in this paper confirm the conclusion of (49). However, the means in age group of 0–2 and 2–5 are quite close to 0, and the paired samples *T*-test results revealed there are no significant statistical differences between the predicted and the actual values on both age groups, which means the predicted results of 0–2 and 2–5 age group are in good agreement with the actual age. Similarly, the predicted results of 0–5 age group are also in good agreement with the actual age according to Figure 7C. Therefore, the predicted results achieved by the proposed CNN-based model are considered to be reliable overall.

Furthermore, the CNN-based methods can achieve more accurate prediction results compared with traditional machine learning methods. Cole et al. (29) reported the detailed comparison between 3D CNN and GPR method in predicting the brain age using different input data (GM, WM, GM+WM, and raw data). We found that the 3D CNN achieved higher performances than the GPR in all kinds of input data in this reference. Especially, the MAE of 4.65 years obtained by 3D CNN are much lower than the MAE of 11.81 years obtained by GPR when the raw data was used as the input.

However, it is not particularly reasonable to compare our results with the above example since the subjects used above are aged 18 to 90 years old. To the best of our knowledge, only two traditional machine learning-based methods for age prediction of young children were investigated currently. Toews et al. (51) firstly developed a feature-based developmental model for predicting infant age using structural brain MR images. They enrolled 92 subjects aged 8–590 days and achieved a MAE of 72 days. Hu et al. (46) proposed a two-stage prediction method named Hierarchical Rough-to-Fine (HRtoF) model for predicting infant age. They enrolled 50 infants aged 14–797 days and achieved a MAE of 32.1 days. Since it is hard to collect the brain images of young children, the data amount reported in (51) and (46) is not large, <100. In our study, we spent over 5 years collecting 220 subjects, which is enough for reaching a convincing conclusion comparing to the above two studies. Table 8 gives the performance comparison of our proposed method and the above two methods. It is found that the proposed 3D CNN gained the best performance in predicting the brain age of infant aged about 0–2 years old. The prediction accuracy of the 3D CNN for the age of 4–1,820 days is even better than the prediction accuracy of Toews's method (51) for the age of 8–590 days.

**Table 8:** Comparison with state-of-the-art approaches.

**Method**	**Subjects**	**Age (days)**	**MAE (days)**
Feature-based developmental model (51)	92	8–590	72
HRtoF model (46)	50	14–797	32.1
The proposed 3D CNN	88	4–697	28.9
The proposed 3D CNN	220	4–1,820	67.6

In addition to traditional machine learning-based methods, we also compared 3D CNN with 2D CNN in predicting brain age of young children using 3D MR images. The inputted 3D images are stacked 2D brain MR images (slices) and there is a gap between two adjacent slices in the actual location of the brain. Thus, we speculated that the correlation between slices will not be great, and we think that 2D CNN model may also be able to complete the age prediction task well with the inputted 3D images. If the prediction effect of the 2D CNN is the same as that of the 3D CNN, then the 2D CNN will be more recommended in clinical practice, because the 2D model requires much less computation and computer memory. However, as Table 7 shows, the proposed 3D CNN outperformed the 2D CNN significantly. This result shows that the small correlation between adjacent slices is beneficial to the prediction accuracy of the model, and also shows the 3D CNN employing 3D kernels is a more reliable resolution that can take all full advantage of spatial contextual information of the 3D MR images for more accurate age prediction (16, 29, 52).

### Predictions for Children Under 2 Years Old Are Better Than Those Over 2

As Table 2 shows, the proposed 3D CNN achieved a MAE of 28.9 days, a RMSE of 37.0 days, a MRE of 7.8%, a *R* of 0.983, and a *R*^2^ of 0.967 in predicting brain age of children aged 0–2 years old, while a MAE of 110.0 days, a RMSE of 133.5 days, a MRE of 8.2%, a *R* of 0.883, and a *R*^2^ of 0.780 were obtained in predicting brain age of children aged 2–5 years old. It is found that the predictions for children under two years old are much better than that over two. Actually, this phenomenon is consistent with the understanding of clinical practice—that is, brain development under 2 years old is rapid and heterogeneous, while the brain over 2 years old develops relatively statically (1, 2). Slow development of the brain over 2 years old leads to low distinguishability and high prediction error.

### Optimizing Model Parameters Can Improve Prediction Accuracy

Generally, the prediction performance is quite dependent on the structure of CNN and the hyper parameters. Thus, we optimized the 3D CNN structure and the hyper parameters with grid search for achieving the best performance on training set and reported the performance on the test set independently. Recently, some evidence reports that network depth is crucially important for achieving remarkable prediction results (53, 54). Thus, we investigated the influence of different network depths on the prediction results, showing in Table 4 and Figure 9. As we can see, the best performance was achieved by the 3D CNN containing seven convolution layers and three fully connected layers, not the deepest or shallowest network. Theoretically, the more convolution layers, the higher the extracted feature levels, and the more fully connected layers, the more complex the mapping function that can be fitted. However, too many neuron layers will produce redundant parameters, easily resulting in overfitting. Except for overfitting, a degradation problem may also occur when the deep network starts to converge: with the network depth increasing, accuracy gets saturated (55, 56). Therefore, in order to obtain the best performance, it is necessary to choose a network structure with the appropriate depth.

If the neural network is too deep, the gradient will become very small when it propagates back to the shallow layer, so that the parameters of the shallow layer cannot be updated or the amplitude of the update is very small. This phenomenon called gradient dispersion will lead to the requirement of lower learning rate and careful parameter initialization. Batch normalization was developed to address the above problems (31). In this study, we firstly tried to set the initial learning rate of the 3D CNN without batch normalization the same as that of the proposed 3D CNN. However, it is found that the learning rate is too high, which leads to the failure of training the 3D CNN without batch normalization. With grid search and trial-and-error methods, we set the learning rate of the network to 0.000000008. This observation fully proves that the network without batch normalization requires more careful parameter setting. According to Figures 5, 10, the training loss of the 3D CNN without batch normalization is bigger than that of the proposed 3D CNN, indicating that the former fits the training data worse than the latter. Furthermore, as Table 5 shows, we observed that batch normalization can greatly improve the prediction performance according to MAE, RMSE, *R*, and *R*^2^. Thus, batch normalization is strongly recommended for use in 3D CNN for predicting brain age using stacked 2D routine clinical brain MR images.

In terms of hyper parameters, we investigated the influence of batch size and learning rate on the performance of the 3D CNN. Basically, the larger the batch size, the more stable the gradient descent and the more accurate the direction. However, large batch size may cause the model to fall into local minimums and cannot come out because of the little noisiness. Small batch size may cause the data distribution to be too random to converge. Thus, the best batch size should be obtained by experiments for making the model converge to the global minimum as much as possible. In this paper, we set the batch size as 16, 32, and 64 for observing their effects on the predictions, showing in Table 6. It is found that the batch size of 64 achieved the best performance. The reason for not increasing the batch size is because the computer does not have enough computing memory, which is also the disadvantage of large size that cannot be ignored. Learning rate controls the convergence speed of model. When the learning rate is set too small, the convergence process becomes very slow and may make the model overfit. When the learning rate is set too large, the gradient may oscillate back and forth around the minimum value, and may not even converge. Thus, it is necessary to select the appropriate learning rate with grid search for achieving the best performance. As Table 6 shows, the middle-sized learning rate yields the best predictions.

## Conclusion

In this paper, we developed an end-to-end AI system based on 3D CNN for predicting the brain age of children aged 0 to 5 years old and achieved reliable and high performance with a MAE of 67.6 days, a RMSE of 96.1 days, a MRE of 8.2%, a *R* of 0.985, and a *R*^2^ of 0.971. We found that the predictions for children under 2 years old are much better than those over two, which is also better than two state-of-the-art methods of predicting brain age of infants. The changes in the structure of the model have small effects on the prediction results, as do the changes in learning rate and batch size. The tricks of data augmentation and batch normalization have a significant impact on model performance. The proposed 3D CNN outperformed the 2D CNN having similar structure in prediction results.

In the future, we will collect more subjects for enhancing the performance of the model since CNN is a kind of data-driven method. Furthermore, we will enroll child patients with neurodevelopmental or mental disorders for validating the performance of the model in predicting the biological age of their brains.

## Data Availability Statement

All datasets presented in this study are included in the article/supplementary material. Code Repository: https://github.com/Captain-Hong/Brain-Age-Prediction-of-Children.

## Ethics Statement

The studies involving human participants were reviewed and approved by Ethics Committee of Children's Hospital of Nanjing Medical University. Written informed consent to participate in this study was provided by the participants' legal guardian/next of kin.

## Author Contributions

ZF and MY collected the data. JH designed the algorithm. JH and Y-DZ preprocessed the data, designed the algorithm, and tested the model. S-HW, MY, and Y-DZ interpreted the results. JH and ZF drafted the work. MY and YZ gave guidance on experiment design. JH, ZF, and S-HW organized the literature. YS and AP substantively revise the manuscript. All authors gave critical comments and approved the submission.

## Conflict of Interest

The authors declare that the research was conducted in the absence of any commercial or financial relationships that could be construed as a potential conflict of interest.
